# Response to the commentary by Melidis *et al.* on “Untargeted CUT&Tag reads are enriched at accessible chromatin and restrict identification of potential G4-forming sequences in G4-targeted CUT&Tag experiments”

**DOI:** 10.1093/nar/gkaf1338

**Published:** 2025-12-12

**Authors:** Alicia K Byrd, Matthew D Thompson

**Affiliations:** Department of Biochemistry and Molecular Biology, University of Arkansas for Medical Sciences, Little Rock, AR 72205, United States; Winthrop P. Rockefeller Cancer Institute, Little Rock, AR 72205, United States; Division of Natural Sciences, Lyon College, Batesville, AR 72501, United States

## Abstract

In their Matters Arising commentary, Melidis *et al.* critically evaluate our work (Nucleic Acids Research, volume 53, gkaf678). In our response, we emphasize why our approach supports the validity of our conclusions. The authors’ suggestion that we failed to include “explicit disclosure of the analytical methods and code” in our original publication is misleading; we used publicly available tools and disclosed explicit parameters and detailed methods for each process in our original publication. While the authors raise several important points, such as the difference in DNA recovery between targeted and untargeted samples, we argue that this difference does not abrogate the need to control for untargeted tagmentation. Due to the co-localization of G4s and preferential Tn5 tagmentation in accessible chromatin, our suggestion that G4 CUT&Tag data be validated is both reasonable and scientifically rigorous.

## Introduction

Tn5-based techniques have become common for mapping of G4-forming loci [1–7]. These methods rely on recognition of a G4 by an antibody or G4 ligand followed by tagmentation by Tn5. Importantly, Tn5 is also used to map accessible chromatin in ATAC-seq [8]. To reduce untargeted Tn5 activity in accessible chromatin during CUT&Tag reactions, a high salt wash is included. However, use of this high-salt wash does not fully abrogate ATAC-seq-like untargeted tagmentation [9, 10], so our study aimed to examine whether open chromatin mapped by untargeted CUT&Tag contributes a significant component of the signal from G4-targeted CUT&Tag. Our efforts are intended to aid others in the field in avoiding possible false positive or false negative identification of G4s, and we hope that our manuscript [11] serves as a helpful resource for the G4-mapping community.

**Figure 1.**
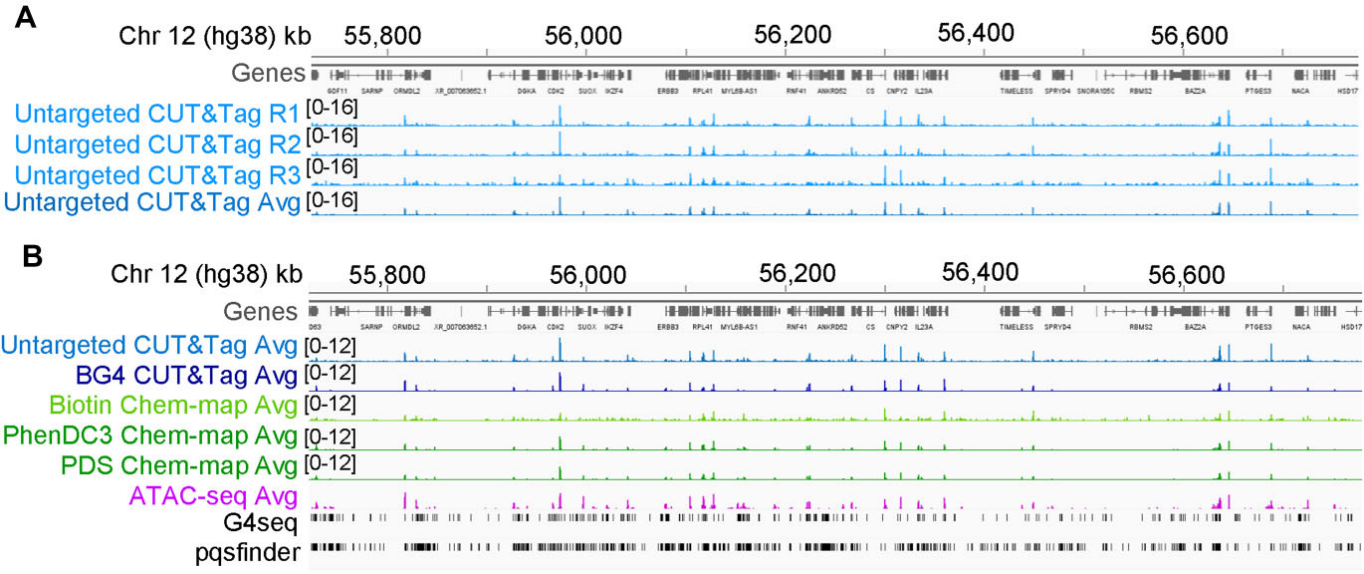
There is a consistent pattern of enrichment at accessible chromatin in untargeted CUT&Tag libraries. (**A**) Sequence coverage of normalized read counts from untargeted CUT&Tag replicates from K562 cells [1] (*n* = 3) plotted individually (R1, R2, and R3) and as the average (Avg) at a representative locus on chromosome 12, following alignment to hg38, deduplication (without downsampling), and normalization with counts-per-million reads (CPM) with the default bin size of 50, as described in our previous work [11]. (**B**) Replicate-averaged untargeted CUT&Tag data as in panel (A) (*n* = 3) and BG4 CUT&Tag data (*n* = 3) from K562 cells [1], Biotin (*n* = 2), PhenDC3 (*n* = 2), and pyridostatin (PDS) (*n* = 2) Chem-map data from K562 cells [3], and ATAC-seq data (*n* = 3) from K562 cells [15] are plotted at a representative locus on chromosome 12, following processing as in panel (A). Additionally, G4s that fold *in vitro* (G4seq) [13] and computationally predicted G4 sequences (pqsfinder) [14] are plotted. All dataset reference information (GEO accessions, etc.) and data processing methods for datasets illustrated in this figure is included in the “Materials and methods” section and Supplementary Table S7 of our prior work [11].

CUT&Tag was originally developed for identification of histone post-translational modifications [9], and no correlation is observed between untargeted CUT&Tag and H3K4me3 CUT&Tag [12]. However, we found there is a significant correlation between the genome-wide signal distribution of untargeted CUT&Tag and G4 CUT&Tag reads [11] with both preferential G4 formation and preferential untargeted Tn5 tagmentation in accessible chromatin, which Melidis *et al.* describe as “unsurprising and inevitable.” This results in clustering of reads from G4-targeted and untargeted CUT&Tag in similar locations; in many cases across various loci throughout the human genome, there is a striking visual similarity between matched G4-targeted and untargeted CUT&Tag signal distributions, as highlighted in our work [11]. Untargeted tagmentation would ideally be eliminated by optimization of experimental conditions. However, as is shown in Fig. 1 of our manuscript [11], despite efforts to eliminate untargeted tagmentation, we observed that a reproducible pattern of genome-wide enrichment is conserved in many of the untargeted datasets, indicating that untargeted tagmentation is often not fully abrogated. Although less DNA is recovered in untargeted CUT&Tag relative to G4-targeted CUT&Tag, “selective enrichment” is not “experimentally absent” (Fig. 1A), as suggested by Melidis *et al.* Reads from replicate untargeted CUT&Tag libraries [1] still exhibit a clear pattern of enrichment and are highly similar across all replicates (Fig. 1A). In Fig. 1B–D of their commentary, Melidis *et al.* highlight biotin ChemMap replicates that have little correlation. We agree that datasets such as these with little enrichment over background exist; indeed, we highlight the SRR14879749 untargeted CUT&Tag dataset in our original manuscript [12]. However, we observed that this is not the case in many of the other untargeted CUT&Tag datasets that we examined, indicating that untargeted tagmentation is often not fully eliminated in practice [12].

Melidis *et al.* suggest that “merging or averaging mapped reads from multiple experiments…can lead to artefacts like an apparent enrichment of background over noise even in untargeted libraries.” We note that much of our analysis in [11] does not use averaged data nor rely solely on conclusions gleaned from this averaged normalized peak count data; the other comparisons in our manuscript [12] rely on datasets using individual replicates, and we only average the normalized read counts for ease of comparison and visualization of multiple datasets ([12] and Fig. 1, this work). Across multiple datasets, we observe that the enrichment in the averaged data matches that in the individual replicates (an example dataset is illustrated in Fig. 1A) and does not “lead to artefacts such as an apparent enrichment of background over noise,” as stated by Melidis *et al.* This reproducible pattern of enrichment in untargeted CUT&Tag libraries persists in individual untargeted libraries from different labs using different cell lines [11]. We believe that the observed correlation between peaks from different datasets that we observe in our work [11], despite additional variables such as cell line of origin, increases the strength of our observation and emphasizes the need to control for this consistent, reproducible genome-wide distribution of untargeted CUT&Tag data at accessible sites. Despite the example posed by Melidis *et al.* in their commentary, the fact that some, but not all, untargeted CUT&Tag libraries have little enrichment over background does not eliminate the need to control for untargeted tagmentation in G4 CUT&Tag as a scientifically sound and broadly applicable consideration when analyzing G4-targeted CUT&Tag data.

We agree with Melidis *et al.* that “the key issue is whether the signal obtained in the presence of a G4 probe can be confidently distinguished from the tagmentation background naturally occurring in its absence.” Published data [1, 3] show that the targeted and untargeted datasets have similar patterns of enrichment at accessible sites (Fig. 1B), indicating that the signal obtained from G4 targeting cannot be readily distinguished from untargeted tagmentation by calling peaks from each distribution and filtering out sites with overlapping peaks. Thus, distinguishing between true signal at a *bona fide* G4 and false positive signal due to untargeted tagmentation may require additional validation.

We agree with Melidis *et al.* that the strong increase in DNA yield from G4-targeted CUT&Tag compared to parallel untargeted CUT&Tag is biologically meaningful and indicates a lack of intended and selective enrichment of the target genomic loci without the primary probe. However, we note that the increased DNA yield in the G4 CUT&Tag experiments compared to untargeted CUT&Tag experiments does not imply, *per se*, a lack of unintended and untargeted tagmentation at a preferred Tn5 site occurring alongside the targeted tagmentation in G4 CUT&Tag experiments. Indeed, it has been documented that untargeted tagmentation contributes a non-negligible number of reads to targeted CUT&Tag libraries [9, 10], and the high degree of overlap between G4s and accessible chromatin could lead to an undesirable degree of untargeted tagmentation occurring alongside targeted tagmentation at G4s within open chromatin that should be accounted for. We note that Melidis *et al.* appear to agree, stating that the “any consensus peaks residually arising from untargeted libraries predominantly correspond to unsuppressed residual Tn5 background activity and will be found in open chromatin regions defined by Tn5-based ATAC-seq experiments.” We emphasize that this “unsurprising and inevitable” co-localization could then lead to calling of false-positive G4s from untargeted tagmentation in accessible chromatin without a folded G4 structure in G4-targeted CUT&Tag reactions under conditions where untargeted tagmentation is not fully abrogated.

Melidis *et al.* also state that the “sparse distribution of sequenced fragments in off-target regions“ and the act of “identifying local enrichment obviates the need for an explicit background sample.” However, we note that the signal is not always sparse and lacking enrichment in untargeted reactions (Fig. 1A and [11]). Additionally, use of an untargeted dataset is recommended with the peak callers for CUT&Tag data [15, 16]. We and others have observed that inclusion of the untargeted control data in peak calling moderately increases the specificity (i.e. precision) but reduces the sensitivity (i.e. recall) of peak calling for CUT&Tag datasets [11, 17]. Thus, it is not surprising that, although it is recommended to use the untargeted CUT&Tag data [15, 16] for peak calling, the untargeted CUT&Tag datasets are not always sequenced, and when sequenced, are not always used in G4 CUT&Tag analysis, as this would lead to *bona fide* G4s being falsely excluded from the set of called G4s.

This omission of untargeted CUT&Tag datasets in peak calling necessitates use of alternative methods to prevent untargeted tagmentation from biasing the output of G4 CUT&Tag. In our work [11], we attempted to use differential enrichment analysis with DiffBind (https://bioconductor.org/packages/devel/bioc/vignettes/DiffBind/inst/doc/DiffBind.pdf) to distinguish between *bona fide* G4s and untargeted tagmentation based on differences in read depth. A similar method had been previously applied for CTCF CUT&Tag [9]. Melidis *et al.* challenge our use of DiffBind due to unequal sequencing depth between the untargeted and targeted CUT&Tag libraries. While we acknowledge the highly discordant sequencing depths between targeted and untargeted CUT&Tag libraries, we argue that the similar signal-to-noise ratio for each library (i.e. FRiP scores) and comparable CPM-normalized read count amplitudes following downsampling and deduplication (as in Fig. 5A [11]) or deduplication without downsampling (as in Fig. 1B, this work) demonstrate that the libraries share sufficient normalized signal distribution to be compared using DESeq2-mediated [18] background bin normalization and relative-log-expression normalization in DiffBind. Importantly, although Melidis *et al.* suggest “over-representation of polymerase chain reaction duplicates” within the untargeted libraries influences our analysis, our analysis avoids this potential confounder by consistent use of deduplication.

Melidis *et al.* also challenge our use of complexity normalization [16] because the increased DNA recovery in the targeted libraries in relation to the untargeted libraries is expected. However, the persistent and reproducible overlap of reads in both complexity-normalized datasets (as in Fig. 5A, [11]) and non-complexity-normalized datasets (as in Fig. 1B, this work) from G4-targeted and untargeted CUT&Tag reactions demonstrates that downsampling does not “skew the real signal distribution,” as stated by Melidis *et al.* While we acknowledge that downsampling could mask subtle differences in the libraries that arise from differences in sequencing depth as suggested by Melidis *et al.*, we note in this response (Fig. 1B) and in our original work that we still observe a substantial read distribution correlation between G4-targeted and untargeted CUT&Tag datasets, despite this vast difference in sequencing depths. We argue that this strengthens our conclusions that these datasets non-randomly overlap, suggesting a consistent and non-random contribution of untargeted tagmentation to libraries produced during G4-targeted CUT&Tag reactions.

Importantly, we reached the same conclusions in our original analysis that did not utilize DiffBind or complexity normalization [19], and the conclusions in our manuscript [11] are not dependent on quantitative measures derived from DiffBind or complexity normalization. Instead, we determined that DiffBind did not aid in distinguishing between *bona fide* G4s and untargeted tagmentation with the parameters used in our analysis [11], and we determined that complexity normalization did not change the outcome of our analyses. Consequently, in our recent work [11], we provided a discussion of other methods of validation with orthogonal methods, such as the elegant G4 deletion control experiments utilized by Melidis *et al.* and colleagues in their recent work [4].

We do agree with Melidis *et al.* that consensus peaks across multiple replicates (both biological and technical) should be considered the true output of the experiment. However, peak calling in the absence of proper negative controls (i.e. untargeted tagmentation) may introduce false-positive peak calls, due to the reproducible overlap of untargeted tagmentation and G4-tagmentation. An alternate method of correction for open chromatin bias in untargeted tagmentation [10], or validation of the results as *bona fide* G4s [4, 6, 11, 20], should be employed to eliminate these potential false-positive peaks.

We also agree with Melidis *et al.* that spike-in controls could be utilized to normalize the read counts between G4-targeted and untargeted CUT&Tag libraries. However, spike-in usage was either unclear or unavailable for many of the datasets we analyzed [11]. We additionally support the intriguing idea raised by Melidis *et al.* that novel spike-ins such as synthetic G4 templates could aid detection of G4s using CUT&Tag-based methods. We also note the recent validation of a novel variation of CUT&Tag that improves resolution for dynamic targets [21], as this could offer unprecedent insight into genome-wide localization of dynamic G4-binding proteins, providing yet another avenue for orthogonal validation of G4 CUT&Tag. We encourage further cooperation, development, and innovation along these lines from researchers in the field, and we welcome detailed documentation of methodological insights and analytical methods to best account for this signal.
